# Changes in the geometry of modern daily disposable soft contact lenses during wear

**DOI:** 10.1038/s41598-021-91779-y

**Published:** 2021-06-14

**Authors:** Patryk Mlyniuk, Joanna Stachura, Alfonso Jiménez-Villar, Ireneusz Grulkowski, Bartlomiej J. Kaluzny

**Affiliations:** 1 Division of Ophthalmology and Optometry, Department of Ophthalmology, Collegium Medicum, Nicolaus Copernicus University, ul. Ujejskiego 75, 85-168 Bydgoszcz, Poland; 2 Institute of Physics, Faculty of Physics, Astronomy and Informatics, Nicolaus Copernicus University, ul. Grudziadzka 5, 87-100 Toruń, Poland

**Keywords:** Medical research, Optics and photonics, Physics

## Abstract

The geometry of contact lenses can be altered by wear but determining the changes that occur in soft contact lenses (SCLs) is challenging. This study aimed to investigate the shape alterations of daily disposable SCLs after wear using swept-source optical coherence tomography (SS-OCT). Forty-five eyes with myopia of − 3.00 diopters (D) were enrolled. The participants wore three types of SCLs: hydrogel lens (nesofilcon A) and silicone hydrogel lenses (delefilcon A and stenfilcon A). The SCLs were scanned 3–6 min after lens removal. We found a significant decrease in the SCL anterior curvature: 0.24 ± 0.17 mm for nesofilcon A, 0.44 ± 0.21 mm for delefilcon A, and 0.53 ± 0.29 mm for stenfilcon A. The changes in the anterior curvature of SCLs correlated moderately with the mean corneal keratometry; Pearson’s correlation coefficients for nesofilcon A and delefilcon A were 0.57 and 0.52, respectively (*P* < 0.001). A statistically significant change in the total diameter was observed in SCL made of stenfilcon A (0.39 mm, *P* < 0.001). To conclude, the central radii of curvature decreased after a wearing period for all three types of daily disposable SCLs to imitate the anterior corneal surface, however, the changes in other geometrical parameters measured with SS-OCT were lens-specific.

## Introduction

Refractive errors are often corrected with soft contact lenses (SCLs). However, despite their many advantages, daily disposable SCLs are not the most frequently chosen lenses by patients due to a lack of awareness of this option or concerns about possible complications^1^. Nevertheless, we have recently observed an overall increase in the usage of daily disposable SCLs due to the lower risk of infection and inflammation compared with extended (sleeping with SCLs) or continuous (using SCLs for thirty days and nights) monthly SCLs. Moreover, daily SCLs are less likely to accumulate deposits of proteins and lipids that impair vision and cause discomfort. Furthermore, apart from using proper hygiene when inserting or removing the contact lenses, patients do not have to perform daily care of their contact lenses^2–4^.

Current SCLs are manufactured with two types of materials: hydrogel and silicone hydrogel. In recent years, continuous development of silicone hydrogel materials have included modification to the lens’s surface to improve patient comfort by increasing material wettability and biocompatibility with a tear film. SCLs material properties such as elasticity, viscosity, and yield strength are determined by the characteristics of the material, including water content and Young’s modulus. The water content of materials can range from 30 to 80%^3,5^. Additionally, Young’s modulus of lens material varies between 0.3 and 1.5 MPa^6^. These material properties allow adjustments of the SCL interface curvature to the shape of the anterior cornea during wear^5–7^. However, little scientific data has been reported regarding whether this change is temporal (only on the eye) or might be present after removal. Geometrical interactions between SCLs and the surface of the eye have a significant impact on the lens performance in terms of comfort and vision quality^8–10^. This knowledge might be useful in the development of new designs and materials for daily disposable SCLs, which are supposed to be the type of lenses most frequently used in the future, especially considering the revolutionary projects of SCLs containing electronic components^11–13^.

Contact lens design is usually described by back optic zone radius, back optic zone diameter, total diameter, back vertex power, or central thickness^1^. Although several techniques to determine the curvatures and shapes of SCLs are available, the measurements are difficult to obtain and have issues with repeatability and reproducibility, mainly due to the instability of SCLs^14^. The radii of curvature can be measured by radiuscope, keratometer, toposcope, microspherometer, and other instruments. The lens diameter can be obtained by projector magnifier, 10 × loupe with graticule, or moiré fringe deflectometer. While the lens thickness can be assessed with an electronic/electrical thickness gauge or radiuscope, the lens power can be measured by focimeter^14^. As shown, measuring all important geometrical parameters of SCLs requires the use of several devices with different specificities.

Optical coherence tomography (OCT) is a modern optical modality that enables visualization (imaging) of contact lenses and facilitates the extraction of quantitative information about the geometric parameters of the contact lenses^15,16^. The newest generation of OCT called swept-source OCT (SS-OCT) utilizes tunable light sources (swept sources) and high-speed detection and acquisition technology^15^. The exceptional performance of SS-OCT in terms of sensitivity and scan speed enables cross-sectional and three-dimensional imaging of semi-transparent objects in a non-contact way.

This study aims to evaluate the changes in geometric parameters of three types of daily disposable SCLs (made of different materials) for low myopia after a whole day of wear using SS-OCT. We applied the original lens auto-positioning method for an SCL immersed in a hyper-reflective water solution to obtain repeatable and comprehensive data on the geometry of SCLs. We also assessed the correlation between the changes in SCL geometry and the shape of the cornea.

## Methods

### Study design

This is a prospective, pre-post interventional study conducted at the Division of Ophthalmology and Optometry, Department of Ophthalmology, Collegium Medicum, Nicolaus Copernicus University in Bydgoszcz (Poland). The study was carried out in accordance with the principles of the Declaration of Helsinki, Good Clinical Practice guidelines, the International Conference on Harmonization, and other applicable laws and regulations. The study was approved by the Ethics Committee on Clinical Investigation at the Nicolaus Copernicus University. Each participant recruited for the study was informed about the nature of the investigation and provided informed consent to participate in the experiments. In the case of a minor, the parents provided informed consent.

### Participants

The research group consisted of 45 eyes and 23 patients, including 37 eyes of women and 8 eyes of men. Only the eyes with a refractive error of − 3.00 diopters (D) were enrolled. The eyes with refractive or corneal astigmatism above 1.50 D were excluded. All participants were experienced contact lens users, without contraindications for wearing SCLs or participating in the study. The baseline characteristics of the enrolled eyes are presented in Table 1.

**Table 1:** Baseline characteristics of enrolled eyes; mean values (standard deviation, SD).

Parameter	Mean (SD), n = 45
Age (years)	23 (14–36)*
Keratometry average (mm)	7.69 (0.17)
Keratometry cylinder (D)	0.91 (0.41)
Horizontal visible iris diameter (mm)	12.2 (0.41)
Corneal sagittal height at corneal diameter (mm)	3.42 (0.26)
Sagittal height of the anterior segment at a chord of 16 mm (mm)	4.51 (0.31)

*Median (range).

### Study protocol

All participants underwent general ophthalmic examination, autokeratometry, and corneal topography/tomography with a Placido/Scheimpflug instrument (Sirius, CSO, Italy). The participants were asked to wear three types of daily disposable SCLs for a minimum of 9 h per day (mean wearing time: 10.4 ± 1.6 h) in random order:

1. nesofilcon A hydrogel lens (Biotrue ONEday, Bausch + Lomb, USA),
2. delefilcon A silicone hydrogel lens (Dailies Total 1, Alcon, USA),
3. stenfilcon A silicone hydrogel lens (MyDay, Cooper Vision, USA).

The participants received the SCLs from investigators. Each SCL used in this study had the power of − 3.00 D. The main characteristics of the daily disposable SCLs used in this study are given in Table 2^17–19^.

**Table 2:** Characteristics of the daily disposable SCLs used in the study^11–13^.

Material	Contact lens		
	Nesofilcon A	Delefilcon A	Stenfilcon A
Trade name	Biotrue ONEday	Dailies Total 1	MyDay
Manufacturer	Bausch + Lomb	Alcon	CooperVision
Diameter (mm)	14.2	14.1	14.2
Base curve (mm)	8.6	8.5	8.4
Central thickness for − 3.00 D (mm)	0.1	0.09	0.08
Refractive index	1.374	1.42	1.401
Young’s modulus (MPa)	0.49	0.7	0.4
Water content	78%	33%	54%

### Custom-made SS-OCT system and SCL measurements

All measurements were performed using the custom-made SS-OCT system operating at the speed of 50 kA-scans/s and a central wavelength of 1310 nm (Axsun Technologies Inc., Billerica, USA). Interference signals were digitized with a dual-channel acquisition board (NI-5762 Digitizer Adapter Module for FlexRIO, National Instruments Corp., Austin, USA, 250 MS/s, 16-bit resolution), enabling a free-space depth (axial) range of 9.5 mm. A sensitivity of 108 dB was measured when the sample was illuminated with a beam of 3.5 mW power. The axial and transverse resolutions were measured to be 9 μm (in air) and 24.8 μm, respectively. We implemented a scanning protocol to obtain 300 B-scans with 300 A-scans to obtain a three-dimensional (3-D) OCT data set.

**Figure 1:**
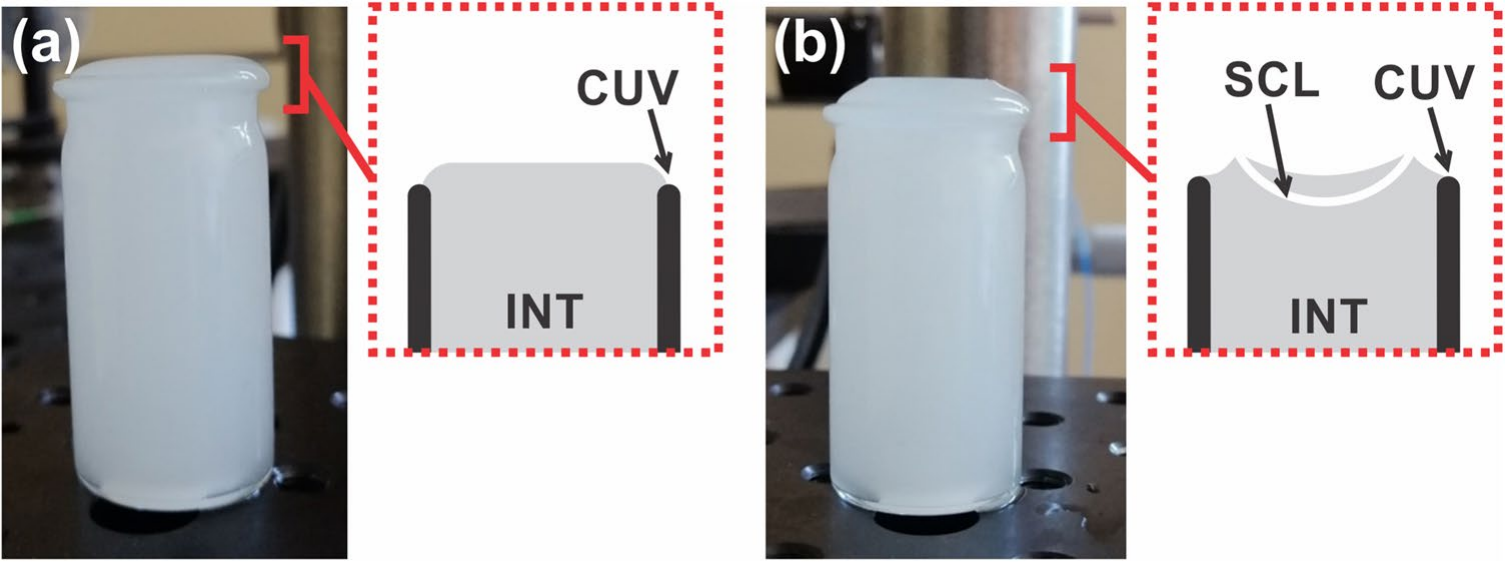
Contact lens measurement with SS-OCT. (**a**) Initial concave meniscus in the wet cell and (**b**) SCL on the concave meniscus. *INT* intralipid solution, *CUV* cuvette wall, *SCL* soft contact lens.

The contact lens was placed in a wet cell filled with 0.5% solution of Intralipid (fat emulsion) and 0.9% saline solution at a temperature of 20 ± 0.5 °C before each scanning session. The auto-positioning method based on lateral capillary forces between floating objects was used. The SCL was placed on the initial convex meniscus with the concave side facing upward. Subsequently, the SCL meniscus was filled with saline solution to prevent the SCL edges from rolling up and to improve edge visualization (Fig. 1). This approach allowed perfect centration of the lens position.

**Figure 2:**
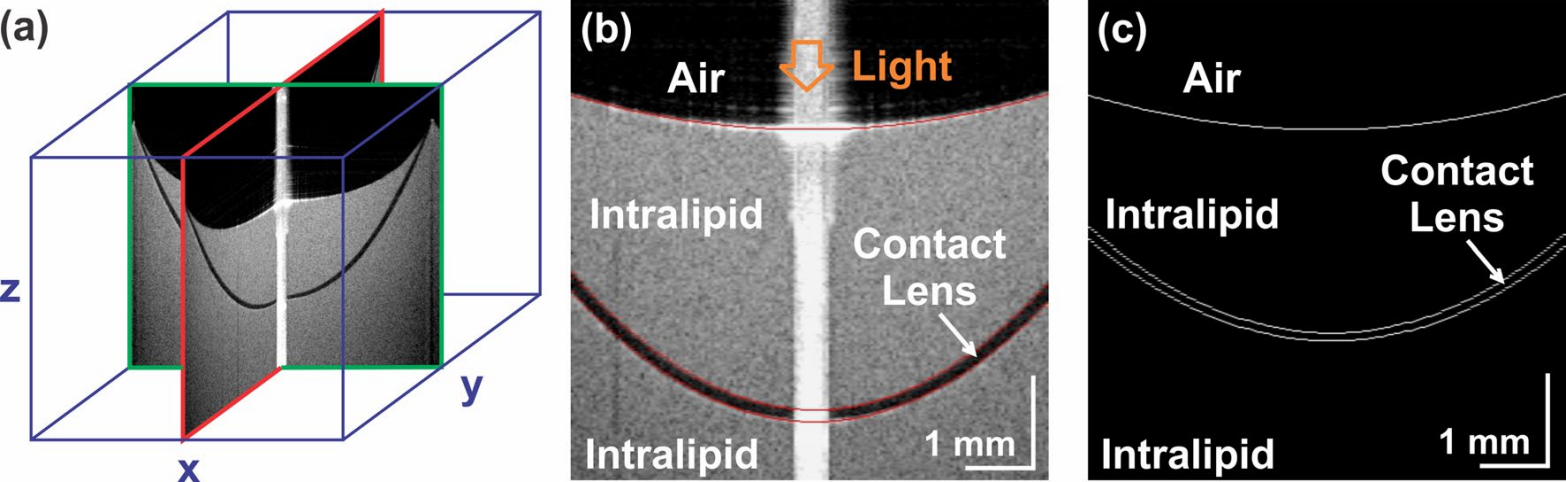
OCT data processing. (**a**) Central cross-sections were extracted from a three-dimensional data set. The lens was then placed in a solution of increased reflectivity. (**b**) The intralipid interface and lens surfaces were segmented (red). (**c**) The images were corrected for light refraction, and profilometric parameters of the lens were measured.

Optimized SCL visualization using a hyper-reflective solution facilitated the extraction of the quantitative parameters. To obtain profilometric information on the measured contact lenses, volumetric 3-D OCT data was processed with the following algorithm (Fig. 2):

- manual segmentation of the Intralipid interface and lens surfaces,
- correction of the cross-sectional images for light refraction using ray tracing,
- extraction of lens parameters: lens thickness, radius of central curvatures, total diameter, and sagittal height.

A best fit spherical surface over a 6 mm diameter central zone was used to calculate the anterior and posterior radii of curvature. Finally, the mean radii values measured in two perpendicular planes (X and Y) were analysed.

First, 5 measurements from 5 different samples of each type of new contact lenses were made to evaluate the repeatability of the method. In this case, the measurements were taken within 3–6 min after removal from the blister pack and placing in a hyper-reflective solution. For each type of SCL, the average of the values served as the baseline for which the impact of wear on lens geometry was compared. The volunteers were asked to wear each SCL for minimum of 9 h per day. The participants were asked to gently remove their contact lenses at the end of the day, and the SCL geometry was measured 3–6 min after removal to allow for the stabilization of the temperature and hydration of each SCL (the same conditions as baseline measurements).

### Statistical analysis

The results for parameters that were normally distributed continuous variables were reported as the mean and standard deviation (SD). For variables that were non-normally distributed, the median and interquartile range (Q1, Q3) were used. The differences between continuous normally distributed variables were analysed using the t-test for independent samples. Repeated measurements variance analysis (ANOVA) was used in the case of dependent samples. When the data was not normally distributed, the differences were compared using the Wilcoxon test (for independent samples) or the Friedman test (for dependent samples). Pearson’s correlation coefficient was calculated to investigate the dependencies between selected continuous variables. When the *P-*value was less than 0.05, the results were considered to be statistically significant. The statistical analysis was performed with R software (v. 3.0.3)^20^.

## Results

The results obtained in this study can be found in Supplementary Table S1. Each parameter was obtained with high repeatability as seen by the narrow standard deviations of each measurement (Table 3). The most important parameters—the central radii of curvature of SCLs—can be determined with precision within the range of 10–70 μm. Mean or median values of five measurements using five different samples of each type of new lenses performed with the same setup and under the same conditions are presented in Table 3.

**Table 3:** Repeatability of measurements of the central radii of central curvature, diameter, sagittal height, and central thickness from new daily disposable soft contact lenses; mean values (standard deviation, SD).

	Nesofilcon A Biotrue ONEday	Delefilcon A Dailies Total 1	Stenfilcon A MyDay
**Radius of curvature (mm)**			
Anterior	8.33 (0.01)	9.64 (0.07)	9.36 (0.01)
Posterior	7.93 (0.01)	9.1 (0.03)	8.85 (0.03)
Diameter (mm)	14.36 (14.36–14.36)*	14.21 (14.19–14.21)*	13.86 (13.85–13.87)*
Sagittal height (mm)	3.64 (3.64–3.65)*	3.8 (3.79–3.8)*	3.62 (3.62–3.63)*
Central thickness (mm)	0.074 (0.001)	0.071 (0.003)	0.059 (0.008)

*Median (range).

**Figure 3:**
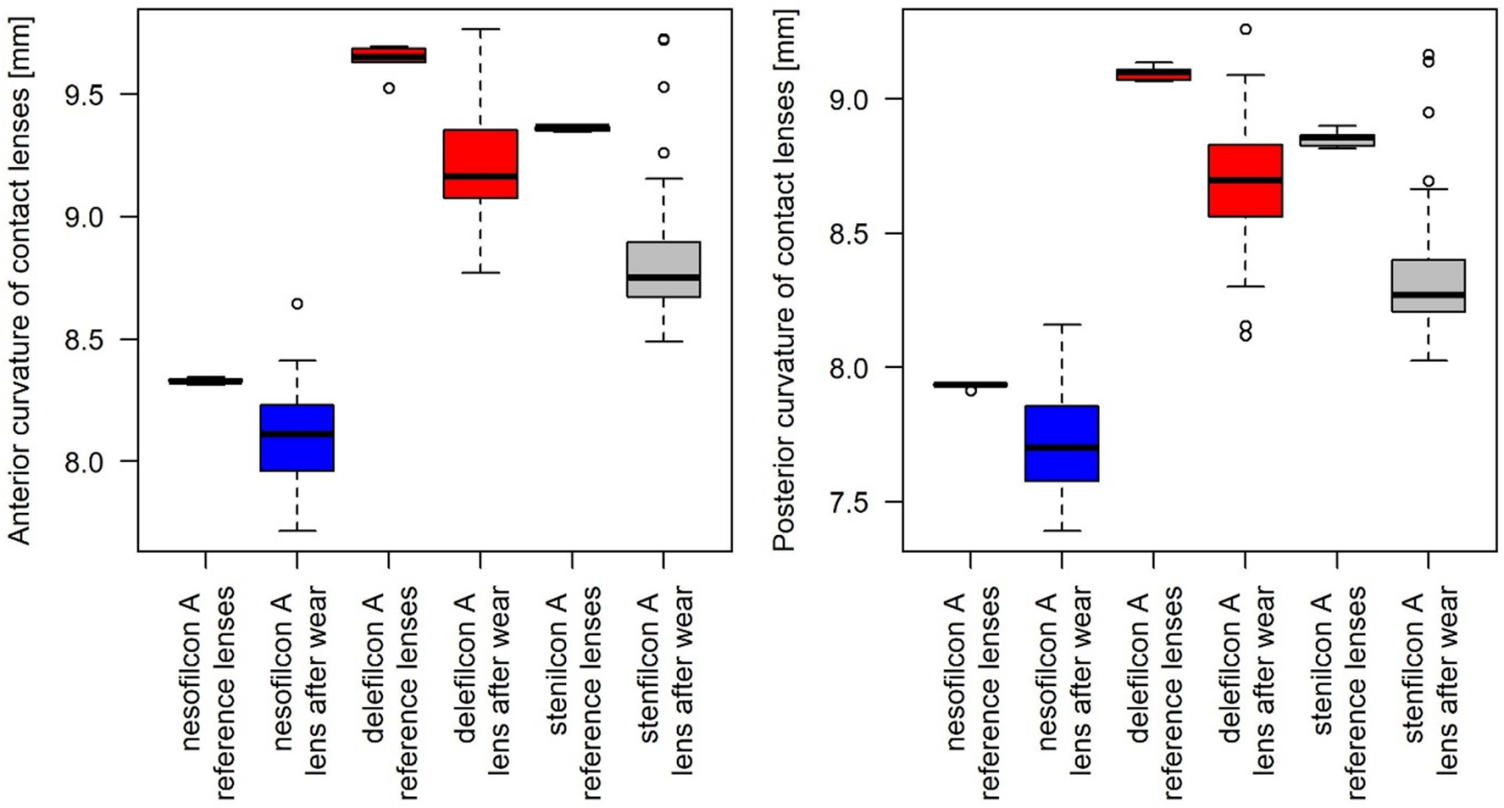
Changes in the anterior and posterior radii of curvature for three types of daily disposable SCLs.

Table 4 presents the changes in the anterior and posterior central radii of curvature for the contact lenses. Both anterior and posterior radii of curvature decreased significantly (*P* < 0.001) for each investigated lens as an effect of wear. Additionally, the changes (Δ) were statistically different between the lenses. The largest decreases in the anterior and posterior radii of curvature were found for the lens made of stenfilcon A, whereas the lowest decreases were observed for SCL made of nesofilcon A. The results included in Table 4 are graphically represented in Fig. 3.

**Table 4:** Changes in total diameter, sagittal height, and central thickness for three material types of daily disposable SCLs; mean values (SD).

	Nesofilcon A Biotrue ONEday				Delefilcon A Dailies Total 1				Stenfilcon A MyDay			
	Before n = 5	After n = 45	∆	*P*	Before n = 5	After n = 45	∆	*P*	Before n = 5	After n = 45	∆	*P*
Anterior radius of curvature (mm)	8.33 (0.01)	8.1 (0.2)	− 0.24 (0.19)	< 0.001	9.64 (0.07)	9.21 (0.21)	− 0.44 (0.21)	< 0.001	9.36 (0.01)	8.83 (0.28)	− 0.53 (0.29)	< 0.001
Posterior radius of curvature (mm)	7.93 (0.01)	7.7 (0.18)	− 0.24 (0.17)	< 0.001	9.1 (0.03)	8.69 (0.23)	− 0.4 (0.22)	< 0.001	8.85 (0.03)	8.34 (0.25)	− 0.51 (0.25)	< 0.001
Total diameter (mm)	14.36 (14.36 to 14.36)*	14.34 (14.25 to 14.38)*	− 0.01 (− 0.12 to 0.03)*	= 0.57	14.21 (14.19 to 14.21)*	14.28 (14.22 to 14.36)*	0.08 (0.02 to 0.15)*	= 0.03	13.86 (13.85 to 13.87)*	14.23 (14.17 to 14.31)*	0.39 (0.32 to 0.46)*	< 0.001
Sagittal height (mm)	3.64 (3.64 to 3.65)*	3.55 (3.52 to 3.59)*	− 0.07 (− 0.12 to − 0.04)*	< 0.001	3.8 (3.79 to 3.8)*	3.73 (3.69 to 3.77)*	− 0.06 (− 0.1 to − 0.02)*	= 0.009	3.62 (3.62 to 3.63)*	3.67 (3.64 to 3.69)*	0.05 (0.02 to 0.07)*	= 0.001
Central thickness (mm)	0.074 (0.001)	0.073 (0.003)	− 0.001 (0.003)	= 0.18	0.071 (0.003)	0.08 (0.069)	0.009 (0.07)	= 0.43	0.059 (0.008)	0.058 (0.003)	− 0.001 (0.003)	= 0.73

*Median (range).

The total diameter, sagittal height, and central thickness were also assessed in this study and the results are summarized in Table 4. The total lens diameter increased for SCLs made of stenfilcon A and delefilcon A: 0.39 mm and 0.08 mm, respectively. On the other hand, the changes in lens diameter were not significant in the case of nesofilcon A. A significant decrease in the value of sagittal height for nesofilcon A and delefilcon A was observed: 0.07 mm and 0.06 mm, respectively. However, the sagittal height increased in the lens made of stenfilcon A, 0.05 mm. We did not observe any significant post-wear change in the central thickness of the SCLs under investigation.

Finally, we examined the correlation between the corneal shape and the change in the anterior and posterior curvatures of the contact lenses (Table 5). For two of the examined SCLs, the changes in the anterior and posterior lens curvature correlated with the mean keratometry value of the cornea (*P* < 0.001). The highest positive correlation for the anterior and posterior lens curvatures was found for the nesofilcon A material. In the case of SCLs made of delefilcon A, a moderate positive correlation between the mean keratometry and a decrease in the radius of curvature was observed. No significant correlation between corneal keratometry and change in lens curvature was obtained for the lens made of stenfilcon A.

**Table 5:** Pearson’s correlation coefficient (r) for the mean keratometry value of the cornea and change in the anterior and posterior curvature of the contact lenses.

	Nesofilcon A Biotrue ONEday		Delefilcon A Dailies Total 1		Stenfilcon A MyDay	
	r	*P*	r	*P*	r	*P*
Anterior curvature	0.57	< 0.001	0.52	< 0.001	0.05	0.77
Posterior curvature	0.56	< 0.001	0.53	<0.001	0.02	0.92

## Discussion

In this study, we took advantage of SS-OCT imaging to investigate post-wear alterations of daily disposable SCLs. In particular, using light scattering medium and SS-OCT it was possible to obtain high-contrast and micrometer-resolution images of the contact lenses. The lens self-centration method based on capillary forces was implemented during the measurement procedure was fundamental in obtaining repeatable quantitative data.

Recently, a device able to visualize and measure the geometry of contact lenses based on spectral-domain OCT was introduced^21,22^. Compared to previous methods, the instrument allows for measuring the sagittal depth and thickness profile of SCLs in addition to the standard geometric parameters of SCLs^21^. This enabled a more comprehensive assessment of changes in the SCLs geometry after wear. However, the effect corneal curvature has on the change in geometry of daily disposable SCLs remains unverified. The SS-OCT prototype used in our study is an example of advanced technology for SCL metrology. The performance of the SS-OCT instrument enables higher sensitivity, lower signal drop with depth, and faster imaging than spectral-domain OCT. Therefore, high-quality and high-resolution data sets are achievable. This feature along with custom-made software allows for highly repeatable measurements of SCL parameters as well as the evaluation of changes occurring secondary to wear. Standard deviations of 5 repeated central radii measurements were within the range of 10–70 μm confirming the high repeatability of this measurement. Low standard deviations within the measurement of 5 samples of control lenses suggested low variability in the production process of moulded soft contact lenses.

There are differences between the measured parameters of new daily disposable SCLs and the specifications given by the manufacturers. Several reasons can be identified to explain these differences. Firstly, different measurement modalities were used to assess the geometry of the SCLs. For example, these parameters can be measured directly from contact lens moulds. Secondly, a hyper-reflective solution, lateral capillary forces, surface tension, and shape distortion may have a minor impact on the shape of the contact lens depending on the material properties, such as elasticity and viscosity. However, it should be noted that the same solution with the same osmolarity was used for both reference SCLs and SCLs after a day of wear. Thirdly, except for the addition of a low concentration of Intralipid (fat emulsion), measurement conditions were in line with ISO standards 18369-3:2017^23^. The total diameter of the SCLs made of nesofilcon A and delefilcon A materials were close to the specifications provided by the manufacturer. A slight difference was observed for SCLs made of stenfilcon A material. When evaluating the base curve of SCLs, the differences in the values obtained in our study in relation to those provided by the manufacturers were relatively high for delefilcon A and stenfilcon A but lower for nesofilcon A. The minor differences in central thicknesses between our results and the manufacturers may be a result of manual segmentation of tomograms.

Our study showed the changes in geometry of daily disposable SCLs depend on the material and the lens design. For example, the smallest changes in curvatures and total diameter occurred in the SCLs made of nesofilcon A material. This may be due to several factors, including lens design, the relatively high central thickness of 0.1 mm, and above all, the fact that it is a hydrogel material without the addition of silicone^17^. Moreover, it may be due to the low elasticity of the material. Another factor influencing the degree of deformation may come from dehydration^24–26^. Morgan and Efron reported a decrease in the total diameter and radius of curvature for hydrogel lenses associating these changes with the dehydration of lenses during wear^27^. Furthermore, the changes in the curvature of SCLs made of nesofilcon A were moderately correlated with the anterior curvature of the cornea.

The changes in the central curvature of SCLs made of silicone hydrogel material delefilcon A moderately correlate with the anterior surface of the cornea. The changes in the curvature of the lens were higher than those for nesofilcon A material. This may be due to the higher elasticity of the delefilcon A material^18^. In turn, similar and even higher changes in the anterior and posterior curvature of the lens can be observed for stenfilcon A, another silicone hydrogel material. This may be due to the higher viscoelastic properties of the material and lower central thickness^19^. In addition, none of the investigated types of daily disposable SCLs showed statistically significant change in central thickness. In the case of delefilcon A, an insignificant increase in central thickness was observed after wear likely due to the hydration gradient, i.e. a variable degree of hydration across the lens section^18^. These SCLs adjust quite well to the cornea, possibly due to repeated tension of the eyelids pushing the lens against the anterior surface of the cornea. Surprisingly, despite large changes in the curvature, there was no correlation with mean corneal keratometry observed. Thus, the change took place regardless of the ocular surface shape.

For stenfilcon A, a significant increase in the total diameter was observed after wear. This might be due to the low elasticity and low thickness^19^. In turn, the other two materials showed a slight increase or stable total diameter after wear. The lenses made of delefilcon A increased in diameter despite the high stiffness of the lens material. This effect may be related to higher hydration of the lens surface layer compared to the hydration of the inner layer (80% versus 33%)^18^. Therefore, since the central, anterior, and posterior radii of curvature for all three types of SCLs decreased during wear, it can be assumed that the peripheral curvature of the SCLs flattens to fit the sclera.

Slight changes in sagittal height for all three types of daily disposable SCLs were observed. Both nesofilcon A and delefilcon A showed decreases in sagittal height whereas stenfilcon A had a minor increase in height. Kempgens and Becker conducted a study that examined the effects of temperature on the sagittal height of the SCLs^28^. The maximum changes in sagittal height occurred at temperatures from 20 to 27 °C and 34 °C and were reported to be ≤ 80 μm. Although the eye surface temperature is 34 °C, the sagittal height of SCLs can be measured at a temperature of 20 °C according to the ISO standard^23,28^. In our study, we obtained changes that ranged from 50 to 70 μm while performing all the measurements at the same temperature. The lenses were measured after a 3–6 min rest in 0.9% saline solution to achieve proper hydration and temperature. Thus, our results should reflect pure geometrical changes only due to the wear.

It should be noted that the changes in the curvature of the SCLs persisted for several minutes after being removed from the eye secondary to the viscoelastic or plastic properties of the materials^26,29^. Pure elastic behaviour would have immediately returned to its initial shape after removal of the SCL from the eye. Almost all soft lens materials show some degree of viscosity in laboratory measurements, however with time constants less than 1 min^5^. We assume that the change in SCLs parameters as a result of wear may be difficult to explain by measurements of new lenses. Plastic behaviour of SCLs observed in this study leads to the changes in SCLs shape during wear, which consequently may affect lens fitting characteristics and dynamics. Thus having a relevant impact on the comfort of SCLs wear^8–10^.

The method applied in this study is limited by the fact that fat emulsion must be added to increase reflectivity during measurements. Although this helps significantly in the visualization of the lens interfaces, it may modify the lens material and lead to changes in shape. The measurement conditions were consistent for new and used SCLs.

## Conclusions

SS-OCT enables the quantitative geometric analysis of SCLs including the parameters such as anterior and posterior curvatures, lens thickness, total diameter, and sagittal height. The implemented measurement procedure with self-centration is suitable for the assessment of SCLs and provides highly repeatable results. The anterior and posterior radii of curvature of all three types of daily disposable SCLs for low myopia decreased during wear with the largest changes occurring in the silicone hydrogel contact lenses irrespective of different Young’s moduli and water content. The changes in the anterior and posterior curvatures demonstrated moderate correlation with the geometry of the anterior surface of the cornea for the contact lenses made of nesofilcon A and delefilcon A. The changes in total diameter and sagittal height were minor and differed according to the particular brand of the lens. No statistically significant changes in the central thickness of the SCLs were observed.

## Supplementary Information

Supplementary Table S1.

## Data Availability

All data generated or analyzed during this study are included in this published article (see: Supplementary Table S1).
